# Diversity and Epidemiology of Mokola Virus

**DOI:** 10.1371/journal.pntd.0002511

**Published:** 2013-10-24

**Authors:** Joe Kgaladi, Nicolette Wright, Jessica Coertse, Wanda Markotter, Denise Marston, Anthony R. Fooks, Conrad M. Freuling, Thomas F. Müller, Claude T. Sabeta, Louis H. Nel

**Affiliations:** 1 Department of Microbiology and Plant Pathology, Faculty of Natural and Agricultural Sciences, University of Pretoria, Pretoria, South Africa; 2 Wildlife Zoonoses and Vector-borne Diseases Research Group, OIE Rabies Reference Laboratory/WHO Collaborating Centre for the Characterization of Rabies and Rabies-related Viruses), Department of Virology, Animal Health Veterinary Laboratories Agency (Weybridge), Addlestone, Surrey, United Kingdom; 3 University of Liverpool, Department of Clinical Infection, Microbiology and Immunology, Liverpool, United Kingdom; 4 OIE Rabies Reference Laboratory/WHO Collaborating Centre for Rabies Surveillance and Research, Friedrich-Loeffler-Institut, Federal Research Institute for Animal Health, Institute of Molecular Biology, Greifswald-Insel Riems, Germany; 5 OIE Rabies Reference Laboratory, Agricultural Research Council, Onderstepoort Veterinary Institute, Pretoria, South Africa; The Global Alliance for Rabies Control, United States of America

## Abstract

Mokola virus (MOKV) appears to be exclusive to Africa. Although the first isolates were from Nigeria and other Congo basin countries, all reports over the past 20 years have been from southern Africa. Previous phylogenetic studies analyzed few isolates or used partial gene sequence for analysis since limited sequence information is available for MOKV and the isolates were distributed among various laboratories. The complete nucleoprotein, phosphoprotein, matrix and glycoprotein genes of 18 MOKV isolates in various laboratories were sequenced either using partial or full genome sequencing using pyrosequencing and a phylogenetic analysis was undertaken. The results indicated that MOKV isolates from the Republic of South Africa, Zimbabwe, Central African Republic and Nigeria clustered according to geographic origin irrespective of the genes used for phylogenetic analysis, similar to that observed with Lagos bat virus. A Bayesian Markov-Chain-Monte-Carlo- (MCMC) analysis revealed the age of the most recent common ancestor (MRCA) of MOKV to be between 279 and 2034 years depending on the genes used. Generally, all MOKV isolates showed a similar pattern at the amino acid sites considered influential for viral properties.

## Author Summary

According to the World Health Organization, rabies is considered both a neglected zoonotic and tropical disease. Among all the lyssavirus species known to exist today, Mokola virus is unique and appears to be exclusive to Africa. In contrast to all other known virus species in the genus *Lyssavirus* of the family *Rhabdoviridae*, its reservoir host has not been identified yet. As only limited sequence information is available, this study significantly contributes to the understanding of the genetic diversity and relatedness of Mokola viruses. In a collective approach, the complete nucleoprotein, phosphoprotein, matrix, and glycoprotein genes of all Mokola viruses isolated to date were sequenced in various rabies laboratories across the world. A phylogenetic analysis was undertaken and the most recent common ancestor was determined. Subsequently, results were linked to epidemiological background data. We also conducted a comparative study of distinct antigenic sites considered influential for viral properties within those genes.

## Introduction

The lyssavirus genus consists of twelve species recognized by ICTV [1] of which five [(Rabies virus (RABV), Lagos bat virus (LBV), Mokola virus (MOKV), Duvenhage virus (DUVV), and Shimoni bat virus (SHIBV)] have been isolated in Africa [2]. LBV, MOKV, DUVV and SHIBV occur exclusively in Africa. SHIBV was recently isolated from *Hipposideros vittatus* (formerly known as *H. commersoni*) [3]. Another proposed lyssavirus species is Ikoma lyssavirus (IKOV) isolated from an African civet in Tanzania [4].

**Figure 1:**
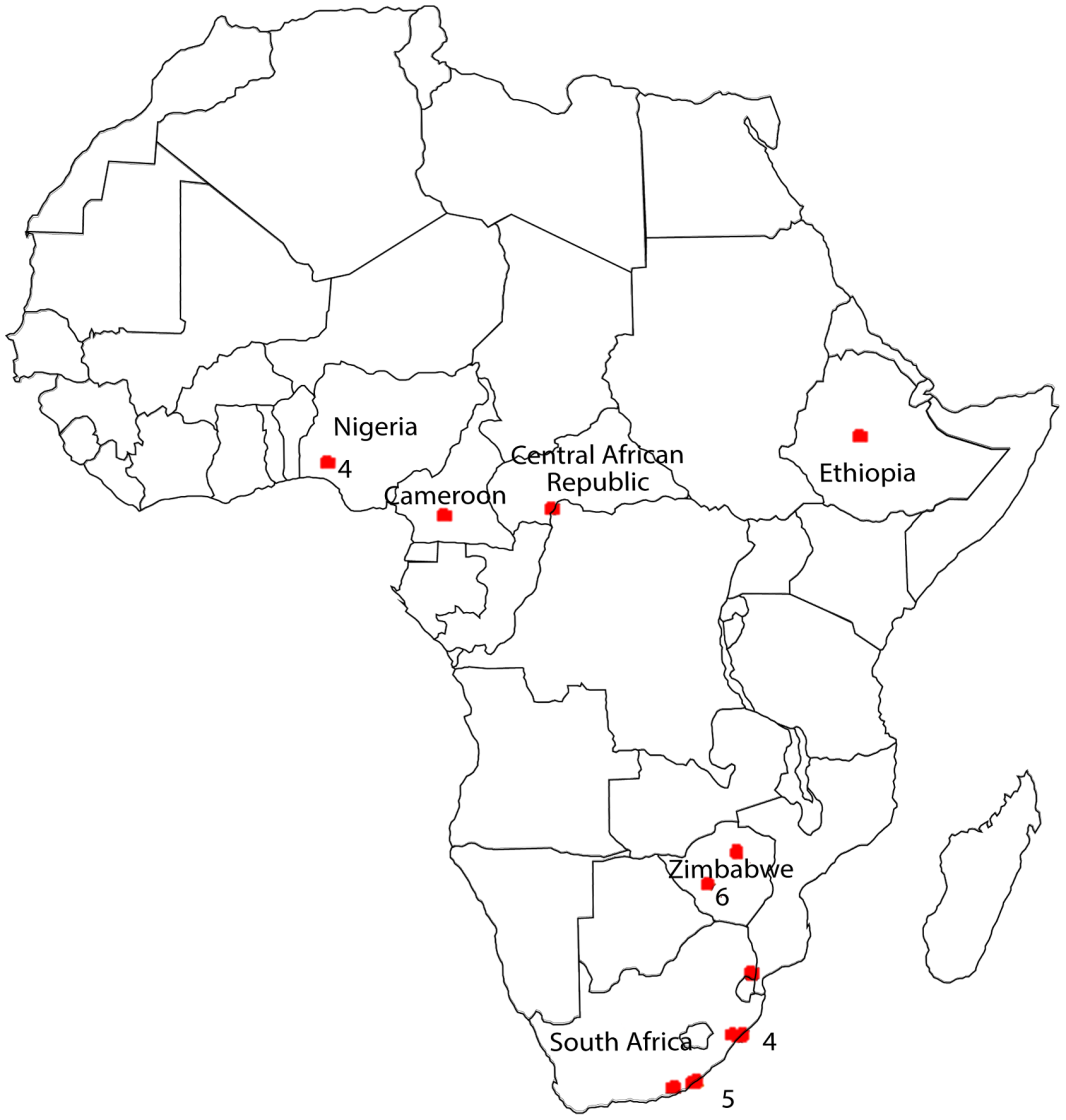
Map of Africa indicating approximate locations of MOKV isolations. The number next to the dots indicates the number of isolates isolated in the same (or in close locations such that the difference cannot be seen in the figure) location.

The first isolations of MOKV were made in 1968 and 1969 from organ pools of shrews (*Crocidura flavescens manni*) in Ibadan, Nigeria [5], [6], [7]. The only isolations from humans were in 1968 and 1971 from two girls from Nigeria [6], [8], [9]. However, there were no classical signs of rabies in either of these cases. Whilst the 1968 isolation was made from the cerebrospinal fluid of a girl who presented with fever and convulsions but fully recovered with no neurological damage, the 1971 isolate was from the brain of a girl who died of a poliomyelitis-like encephalitic disease. A further isolation was made in 1974 from a shrew (*Crocidura* spp.) in Yaounde, Cameroon [10]. The only isolation from a rodent (*Lophuromys sikapusi*) was in 1981, from Bangui, Central African Republic [11]. MOKV was also isolated from other animal species including companion animals. A survey on lyssaviruses undertaken in Zimbabwe between 1981 and 1984 revealed six isolations of MOKV from domestic animals, namely a dog and cats that had been previously vaccinated against rabies and unvaccinated cats [12], [13]. In 1989 MOKV was isolated from a cat in Addis Ababa, Ethiopia [14]. No further isolation of MOKV was made in Zimbabwe until 1993, when the virus was again isolated from a domestic cat [15]. In the Republic of South Africa, the first isolation was made in 1970 from a domestic cat in Umhlanga Rocks, Kwa-Zulu Natal Province (KZN) [16]. At the time the isolate was assumed to be RABV and the isolate was only identified retrospectively using antigenic typing with monoclonal antibodies during the discovery of MOKV in Zimbabwe in the 1980s [17]. Twenty five years later, in 1995, MOKV was isolated from a domestic cat in South Africa, this time from Mdantsane in the Eastern Cape Province (EC) [17]. Two more isolations followed in 1996, one each in KZN and EC and both from domestic cats of which one was vaccinated against rabies [18], [19]. In 1997 and 1998 three more isolations were made from rabies-vaccinated cats in KZN [18], [19]. Following several years in which MOKV was not encountered, two isolations were from rabies-vaccinated domestic cats in 2006 and 2008 from the EC province and these are the most recent known isolations of this virus [20], [21]. From South Africa all isolations of MOKV were from a domestic cat. Viral RNA was detected by PCR from a domestic dog (in 2005) from Mpumalanga Province of South Africa, virus isolation was unsuccessful in this case [20]. A summary of all MOKV isolates and the approximate geographic location of their origin are presented in Table 1 and Fig. 1. Generally, MOKV infected domestic animals were not observed to be particularly aggressive, but displayed other rabies-like signs that included dehydration, unusual behavior, hypersensitivity, neurological disturbance and salivation [19]. Despite MOKV being isolated from a variety of mammal species, this species is the only lyssavirus never to have been isolated from bats.

**Table 1:** Mokola virus reports from 1968–2012.

Geographical location	Year	Origin	Lab reference number	Reference	Accession numbers
Ibadan, Nigeria	1968	Shrew (*Crocidura spp*)	RV4	[7], [8]	KF155005
Ibadan, Nigeria	1968	Human		[7], [10]	?†
Ibadan, Nigeria	1969	Shrew (*Crocidura spp*)		[7]	?†
Umhlanga Rocks, KwaZulu Natal Province, South Africa	1970 (Identified in the 1980's)	Feline	700/70	[17]	FJ465416 (N), AF049118 (P), GQ472989 (M), GQ473001 (G)
Ibadan, Nigeria	1971	Human		[7], [9]	?†
Yaounde, Cameron	1974	Shrew (*Crocidura spp*)	RV39	[11]	#EU293117
Bangui, Central African Republic	1981	Rodent (*Lophuromys sikapusi*)	RV40	[12]	EU293118
Bulawayo, Zimbabwe	1982	Feline	13270	[13]	KC218932 (N), GQ500114 (P), GQ472990 (M), GQ473002 (G)
Bulawayo, Zimbabwe	1981	Feline	12341	[13]	FJ465417 (N), GQ861350 (P), GQ472991 (M), GQ473003 (G)
Bulawayo, Zimbabwe	1981	Feline		[51]	NC_006429
Bulawayo, Zimbabwe	1981	Feline	12574	[13]	FJ465418 (N), GQ861352 (P), GQ472994 (M), GQ473004 (G)
Bulawayo, Zimbabwe	1982	Feline	Zim82/RV1035	[13], [31]	KF155006
Bulawayo, Zimbabwe	1981	Canine (vaccinated)		[13], [31]	?†
Addis Adaba, Ethiopia	1989–1990	Feline	RV610	[15]	#AY333111 (N)
Selous, Zimbabwe	1993	Feline	21846/RV1017	[16]	KC218933 (N), GQ500115 (P), GQ472993 (M), GQ500109 (G)
Mdantsane, Eastern Cape Province, South Africa	1995	Feline	543/95	[18]	FJ465415 (N), GQ500116 (P), GQ472992 (M), GQ500110 (G)
East London, Eastern Cape Province, South Africa	1996	Feline	112/96/RV1021	[19], [20]	KF155008
Yellow Sands, Eastern Cape Province, South Africa	1996	Feline (vaccinated)	322/96	[19], [20]	FJ465414 (N), GQ861353 (P), GQ472996 (M), GQ500111 (G)
Pinetown, KwaZulu Natal Province, South Africa	1997	Feline (vaccinated)	252/97	[19], [20]	JN944637 (N), AF369376 (P), GQ472997 (M), GQ500112 (G)
Pinetown, KwaZulu Natal Province, South Africa	1997	Feline (vaccinated)	229/97	[19], [20]	FJ465413 (N), AF369375 (P), GQ472998 (M), GQ500113 (G)
Pietermaritzburg, KwaZulu Natal Province, South Africa	1998	Feline (vaccinated)	071/98	[19], [20]	FJ465410 (N), AF369378 (P), GQ473000 (M), GQ500108 (G)
Nkomazi, Mpumalanga Province, South Africa	2005	Canine	404/05	[21]	?†
East London, Eastern Cape Province, South Africa	2006	Feline (vaccinated)	173/06	[21]	FJ465412 (N), GQ861351 (P), GQ472999 (M), HQ266624 (G)
Grahamstown, Eastern Cape Province, South Africa	2008	Feline (vaccinated)	226/08	[21]	KC218934 (N), KC218935 (P), KC218936 (M), KC218937 (G)

† Indicates that the existence of the isolate is not known and no full gene sequences were available in Genbank or

# the sequences were likely the same isolate and therefore were not included for MCC analysis.

Cross protection of WHO and OIE recommended rabies vaccines against various rabies-related lyssavirus species have been reported in a number of studies [22], [23], [24], [25], [26]. However, no rabies vaccine provided complete protection against MOKV [27], [28], [29], [30], [31], [32]. More evidence that RABV derived vaccines do not protect against MOKV infection is shown by circumstantial evidence of the fatal infections of numerous domestic animals that had been vaccinated against RABV [13], [18], [19], [20], [21]. Given this scenario and the apparent obscurity of MOKV, we argue that much more information is needed to improve our scant understanding of the epidemiology, disease dynamics and the ecology of this virus.

Some phylogenetic studies have been undertaken on MOKV [18], [20], [21], [33], [34], but these studies were invariably performed on a smaller number of isolates and limited to partial gene sequences. Despite these limitations, these studies provided some evidence of the existence of different virus clusters, delineated according to geographical incidence. Generally, the genetic variance was shown to be inversely related to the spatial distribution of isolates. For example, South African MOKV isolates were shown to be closely related, but distinguishable based on province and as a cluster more distant from those made in a neighboring country, Zimbabwe [21], [33], [34]. Such patterns of genetic diversity may indicate extended periods of isolated evolution, as have been reported for terrestrial rabies virus variants [35]. An exception appeared to be a grouping that included one isolate from Cameroon and one from Ethiopia.

The study reported here involved a multi-disciplinary collaborative effort amongst various laboratories in order to generate for the first time a comprehensive dataset of all the known MOKV isolates available. We have shown that most, but not all of the viruses mentioned in literature could be tracked and that some contamination or misnaming occurred. Given a final cohort of eighteen MOKV isolates, the objective of the study was to sequence full nucleoprotein (N), phosphoprotein (P), matrix (M) and glycoprotein (G) genes. The estimation of viral lineage divergence times and subsequent application of a molecular clock is dependent on an accurate estimation of the rate of nucleotide substitution. Bayesian techniques using the Markov Chain Monte Carlo (MCMC) methods have been successfully applied to estimate the evolutionary rate and divergence times from dated sequences of RABVs [36], [37], [38], [39], [40]. This study applied a relaxed molecular clock to N-, M-, P- and G-gene datasets to obtain estimates of the time to the most recent common ancestor (MRCA) and rate of evolution for MOKV. The subsequent analysis allowed for study of the phylogeny and diversity within this African lyssavirus species.

## Materials and Methods

### Virus isolates

MOKV included in this study were comprised of archived isolates. Information on the geographic location, year of isolation, species origin and references of those MOKV isolates is presented in Table 1. The isolates were either passaged several times (passage number unknown) in suckling mice or in tissue culture, or both. Total RNA was extracted from the samples using the TRIzol® method (Invitrogen) according to the manufacturer's instructions.

### Primer design, RT-PCR and sequencing

The N, P, M and G genes were sequenced using different primer combinations and cycling conditions available from the authors upon request. All PCR products were analyzed by agarose gel electrophoresis and subsequently purified (Wizard PCR Preps DNA Purification System; Promega). The purified PCR products were sequenced with BigDye Termination Cycle Sequencing Ready Reaction Kit 3.1 (Applied Biosystems) according to the manufacturer's protocol and analyzed on an ABI Prism 3100 DNA sequencer (Applied Biosystems). Within the duration of this project next generation sequencing technology became available and was applied on a selection of samples. Complete genome sequence was obtained directly from brain tissue for four MOKV isolates (RV4, RV1017, RV1021 and RV1035) (Marston, unpublished). Briefly, TRIzol (Invitrogen) extracted viral RNA was depleted of host genomic DNA using RNase-free DNAse (Qiagen, UK) and host ribosomal RNA was depleted using Terminator 5′-Phosphate-Dependent Exonuclease (Epicentre Biotechnologies). The RNA was fragmented, a random-primed cDNA library was made and run using the Roche 454 GS FLX System. The sequencing data were assembled in the GS de novo assembly software (Roche). The de novo assembled contigs for each isolate were individually aligned using Seqman (DNAStar) using reference sequence EU293117 and/or specific isolate sequences where available. The resulting consensus sequences were used in GS Reference Mapper (Roche) to obtain further sequence reads from the original raw data for each isolate. All four complete genome sequences were obtained, apart from the extremities of the genome (UTRs). The UTRs were inferred from the previously determined MOKV UTR sequences by using RT-PCR primers situated at the beginning and end of the genome (Marston, unpublished).

### Phylogenetic analyses

Nucleotide sequences were assembled and edited using Vector NTI 9.1.0 (Invitrogen). Multiple sequence alignments were generated using ClustalX and exported in FASTA format. Phylogenetic and evolutionary analyses were conducted using Mega 5.05 [41] for a variety of data sets, i.e. the N, P, M and G gene nucleotide sequences as well as the concatenated sequence. The p-distances between MOKV N gene nucleotide and amino acids sequences were also calculated.

The Maximum Clade Credibility (MCC) phylogenetic tree, estimates of the rate of molecular evolution (substitutions per site per year) and the most recent common ancestor (MRCA) for MOKV were inferred using a Bayesian Markov Chain Monte Carlo (MCMC) method in the BEAST package (BEAST and associated programmes are available via http://beast.bio.ed.ac.uk/) [42]. For this analysis, an input file for BEAST was generated using the BEAUti programme. For MCMC analysis of the concatenated gene sequence dataset (N, P, M and G genes) partitioning into genes was implemented The analysis utilized the general time reversible model with gamma distribution and proportion of invariable sites (GTR+G+I) with site heterogeneity [43] and population histories were constructed using the Bayesian skyline plot [44]. The relaxed (uncorrelated lognormal) molecular clock was chosen as demographic model. The statistical uncertainty in the data for each parameter estimate is reflected by the value of the 95% highest posterior density (HPD). For each estimate, duplicate BEAST runs were performed to test the reproducibility of the analysis. The BEAST output was assessed using the TRACER programme. For each analysis, a chain length of >30 million steps resulted in an effective sampling size (ESS>200 unless noted), with 10% burn-in removed. Trees and parameters were recorded every 10 000 steps. The trees obtained from BEAST were used as input for the TREEANNOTATOR programme to find the MCC tree. Phylogenetic trees were edited for publication using FigTree (version 1.3.1; http://tree.bio.ed.ac.uk/software/figtree/) [42]. Posterior probability values represent the degree of support for each node on the tree.

## Results

**Figure 2:**
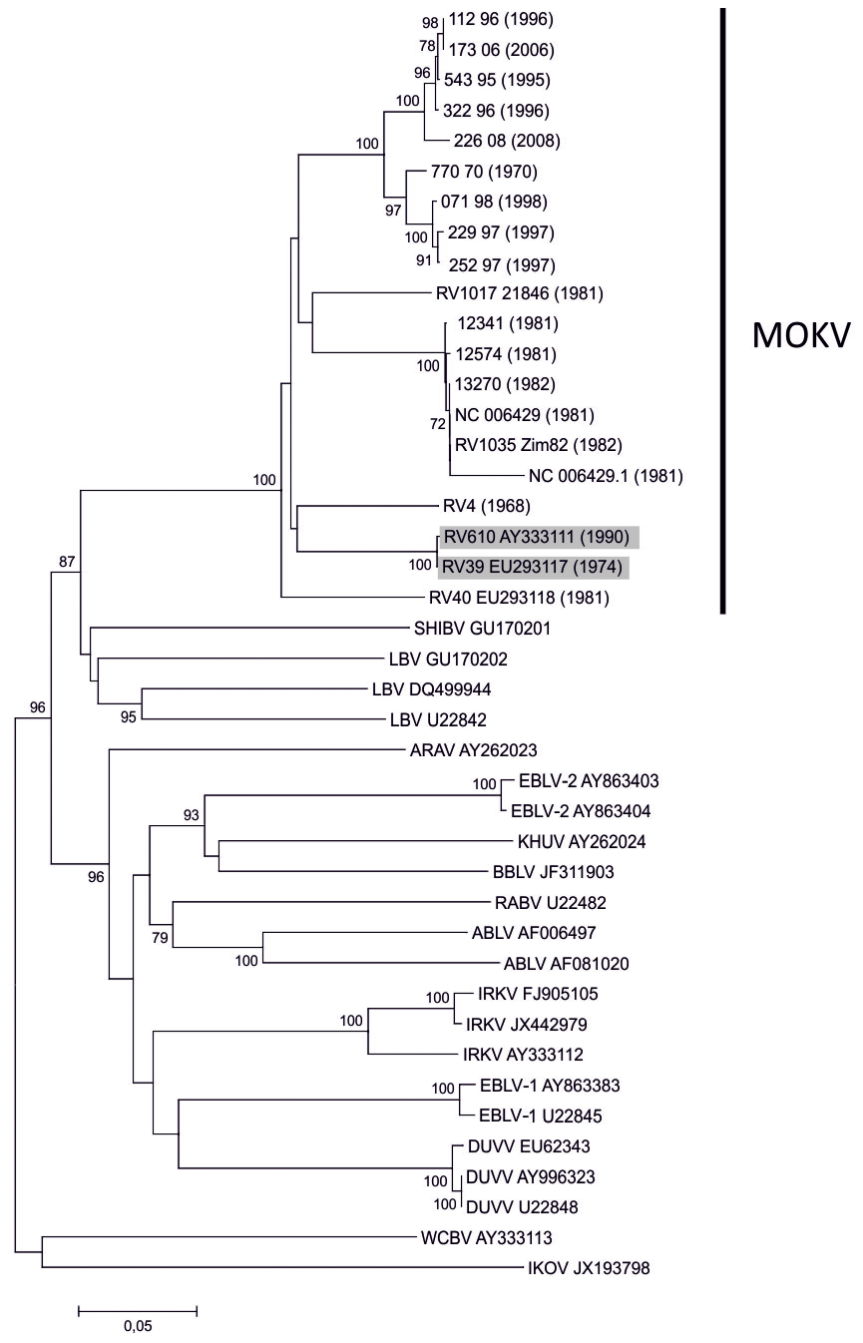
Evolutionary relationships of lyssaviruses inferred using the Neighbor-Joining method (500 replicates) using the Kimura 2-parameter method.

We have generated a comprehensive dataset of all available isolates of MOKV, however, we were unable to trace some, as indicated in Table 1. Of the 24 reported detections of MOKV over the past 50 years, only 18 isolates could be included in this study. We were unable to track virus isolates for four cases reported in literature. Three of these were historical cases from Nigeria, the existence of which are now uncertain viz. two human isolates and a further isolate from a shrew [6], [8], [9]. The fourth was a dog-associated case reported in recent times from South Africa [20], for which an isolate was never produced. Isolates RV39 (Cameroon, 1974) and RV610 (Ethiopia, 1990) were excluded from the MCC phylogenetic analyses as sequence data indicated that these isolates, in our hands, were likely to be the same virus (Fig. 2). A small number of nucleotide differences on some genes were believed to be due to mutations introduced from multiple cell culture passages over years of laboratory maintenance.

A number of domains on the lyssavirus genome have been implicated in the varying degrees of virulence between virus isolates of a lyssavirus species, as well as between virus isolates of different species of the *Lyssavirus* genus. A comparison of these amino acid positions is provided in Table 2. A similar pattern of amino acid substitutions on these positions was observed for the majority of MOKV isolates with specific differences observed on AA 144 (P-gene), AA 81 (M-gene) and AA 194, 198, 268, 352 and 330 (G-gene).

**Table 2:** Comparison of amino acid (AA) sites of MOKV isolates influential for viral properties.

protein	region	Function/Effect	most common motive	deviations (isolate)	reference
N	AA 273	evasion of retinoic acid-inducible gene I mediated innate immunity and pathogenicity	F		[60]
	AA 394				
P	AA 144-148	P protein binding to the LC8 dynein light chain	IQIQT	VQIQT (229-97; 071-98; 252-97; 770-70)	
M	AA 22-25	important for pathogenicity of a related rhabdovirus,VSV	A		[61]
	AA 35-38	efficient virion release and pathogenicity	PPEYVPL		[62]
	AA 77	important in disruption of the mitochondrion and induction of apoptosis	K		
	AA 81		N	S (071-98; 770-70; 229-97; 252-97)	[63]
	AA 95	Val to Ala at position 95 results in increased apoptosis	V		[64]
G	AA 194	Asn – Lys increased viral spread, internalization & pathogenicity	S		[65]
	AA 198	mutation of Arg/Lys 198 resultsin reduced pathogenicity	K	Q (RV4; EU293118)	[66]
	AA 242	important for pathogenicity of the Nishigahara strain (Ile 268 most important residue)	S		[67]
	AA 255		N		
	AA 268		I	V (RV1017)	
	AA 318	p75NTR receptor binding	L		[68]
	AA 352		M	L (226-08)	
	AA 330-333	Arg/Lys 330 responsible for virulence in mice	KRVD	NRVD (RV1017)	[69], [70]
		Double mutation R/K 333 and R/K 330 further reduces virulence			[71]

**Figure 3:**
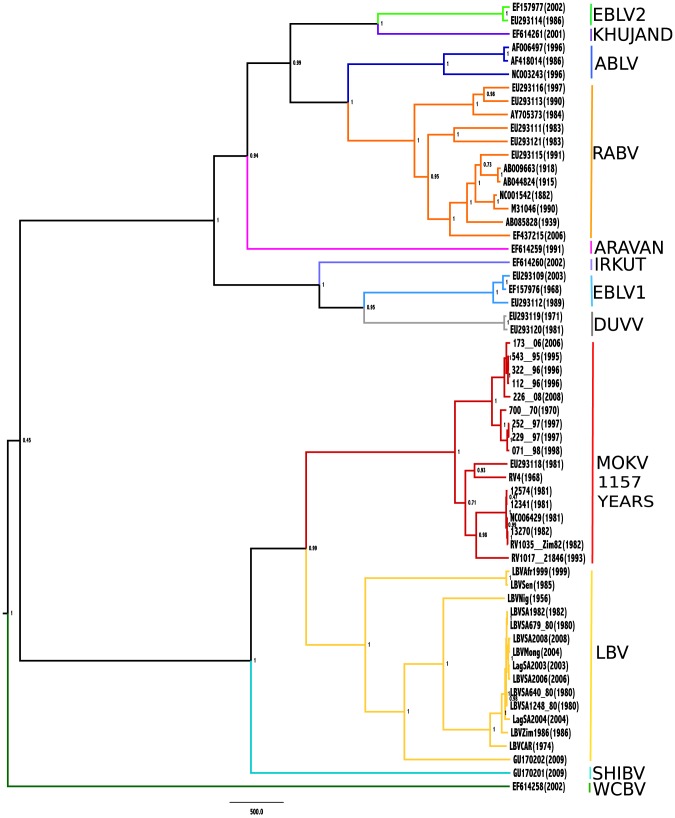
MCC phylogenetic tree based on the concatenated nucleotide sequence of the complete N, P, M and G gene of MOKV isolates and representative lyssavirus isolates. A table indicating the details of the isolates used in the analysis is provided in the supplementary material (Table S1).

The genetic relationships between the different MOKV isolates was determined by construction of a MCC tree using the concatenated full coding regions of the N, P, M and G gene sequences as well as the individual genes (supplementary material). The MOKV isolates analyzed in this study formed a cluster supported by bootstrap values >70% when nucleotide sequences from the concatenated N, P, M and G genes (Fig. 3), N (supplementary material, Fig. S1), P (supplementary material, Fig. S2), M (supplementary material, Fig. S3) and G gene (supplementary material, Fig. S4) were used. The same tree topology was observed for both nucleotide and amino acids sequence analysis (data not shown). The MCC trees indicated that isolates grouped according to geographic location. Phylogenetic analysis of MOKV isolates from South Africa and Zimbabwe demonstrated geographic clustering consistent with previous findings [18], [34]. The South African isolates formed two clusters consisting of KZN and EC provinces respectively. The Zimbabwean isolates from the 1980s (all from Bulawayo) formed a single cluster, distinct from the single 1993 isolate (from Selous). The same grouping was demonstrated for these isolates (South African and Zimbabwean) irrespective of the gene used for phylogenetic analysis.

The Central African Republic and Nigeria isolates formed independent clusters irrespective of the gene used for analyses.

The P-distance comparison between different MOKV isolates was performed using the N gene nucleotide and amino acid sequences (Table 3). Comparison of the nucleotide sequences indicated the difference between the MOKV isolates to be between 0 and 15% (85% nt seq identity), with the highest value (15%) being between U22843 (Zimbabwe) and RV4 (Nigeria). The highest nucleotide difference between South African isolates was 5.7% (226/08 and 229/97) while for Zimbabwean isolates it was 12.3% (between U22843 and RV1017/21846). Collectively, the nucleotide difference between South African isolates and Zimbabwean isolates was 14.4% (U22843 and 226/08). When comparing amino acid differences between MOKV isolates the same trend was observed, with MOKV isolates displaying an overall intragenotypic amino acid variation of 6.4%.

**Table 3:** Percentage difference of the nucleotides (A) and amino acids (B) of the N protein of MOKV isolates.

A	LBVSA2008	071/98	252/97	229/97	700/70	112/96	173/06	543/95	226/08	322/96	U22843	12341	12574	NC_006429	13270	RV1017/21864	EU293118	RV4	RV1035/Zim82
LBVSA2008																			
071/98	0.224																		
252/97	0.224	0.004																	
229/97	0.227	0.007	0.003																
700/70	0.226	0.021	0.023	0.023															
112/96	0.223	0.048	0.049	0.050	0.036														
173/06	0.224	0.050	0.050	0.052	0.037	0.010													
543/95	0.224	0.045	0.047	0.049	0.033	0.003	0.007												
226/08	0.224	0.053	0.056	0.057	0.041	0.018	0.019	0.015											
322/96	0.224	0.046	0.048	0.050	0.033	0.005	0.010	0.002	0.017										
U22843	0.239	0.136	0.138	0.138	0.135	0.139	0.142	0.141	0.144	0.139									
12341	0.213	0.136	0.110	0.110	0.107	0.112	0.115	0.113	0.116	0.111	0.032								
12574	0.214	0.110	0.111	0.111	0.109	0.115	0.116	0.115	0.118	0.113	0.033	0.003							
NC_006429	0.215	0.110	0.111	0.111	0.109	0.113	0.116	0.115	0.118	0.113	0.030	0.001	0.003						
13270	0.215	0.110	0.111	0.111	0.109	0.113	0.116	0.115	0.118	0.113	0.030	0.001	0.003	0.000					
RV1017/21846	0.226	0.104	0.105	0.107	0.096	0.108	0.107	0.107	0.108	0.106	0.123	0.099	0.099	0.099	0.099				
EU293118	0.220	0.117	0.116	0.119	0.116	0.118	0.119	0.118	0.118	0.117	0.145	0.118	0.119	0.119	0.119	0.111			
RV4	0.224	0.110	0.111	0.113	0.107	0.113	0.112	0.112	0.117	0.111	0.150	0.124	0.124	0.124	0.124	0.112	0.111		
RV1035/Zim82	0.215	0.110	0.111	0.111	0.109	0.113	0.116	0.115	0.118	0.113	0.030	0.001	0.003	0.000	0.000	0.099	0.119	0.124	

B	LBVSA2008	071/98	252/97	229/97	700/70	112/96	173/06	543/95	226/08	322/96	U22843	12341	12574	NC_006429	13270	RV1017/21864	EU293118	RV4	RV1035/Zim82
LBVSA2008																			
071/98	0.107																		
252/97	0.107	0.000																	
229/97	0.109	0.002	0.002																
700/70	0.104	0.007	0.007	0.009															
112/96	0.109	0.013	0.013	0.016	0.007														
173/06	0.109	0.013	0.013	0.016	0.007	0.000													
543/95	0.109	0.013	0.013	0.016	0.007	0.000	0.000												
226/08	0.113	0.018	0.018	0.020	0.011	0.004	0.004	0.004											
322/96	0.109	0.013	0.013	0.016	0.007	0.000	0.000	0.000	0.004										
U22843	0.147	0.058	0.058	0.060	0.053	0.060	0.060	0.060	0.064	0.060									
12341	0.102	0.013	0.013	0.016	0.009	0.016	0.016	0.016	0.020	0.016	0.044								
12574	0.102	0.013	0.013	0.016	0.009	0.016	0.016	0.016	0.020	0.016	0.044	0.000							
NC_006429	0.102	0.013	0.013	0.016	0.009	0.016	0.016	0.016	0.020	0.016	0.044	0.000	0.000						
13270	0.102	0.013	0.013	0.016	0.009	0.016	0.016	0.016	0.020	0.016	0.044	0.000	0.000	0.000					
RV1017/21846	0.107	0.013	0.013	0.016	0.011	0.018	0.018	0.018	0.022	0.018	0.053	0.009	0.009	0.009	0.009				
EU293118	0.107	0.011	0.011	0.013	0.013	0.016	0.016	0.016	0.020	0.016	0.056	0.011	0.011	0.011	0.011	0.011			
RV4	0.107	0.020	0.020	0.022	0.018	0.020	0.020	0.020	0.024	0.020	0.064	0.020	0.020	0.200	0.200	0.020	0.018		
RV1035/Zim82	0.102	0.013	0.013	0.016	0.009	0.016	0.016	0.016	0.020	0.016	0.044	0.000	0.000	0.000	0.000	0.009	0.011	0.020	

In order to investigate the evolutionary relationship of MOKV, a MCMC analysis was used to estimate the rate of nucleotide substitution calculated in substitutions/site/year as well as the time of the most recent common ancestor (MRCA) of MOKV (Table 4). When analyzing the N and G gene datasets, the mean nucleotide substitution rate was estimated to be 2.172×10^−4^ (N) and 2.123×10^−4^ (G). This is in agreement with previously published nucleotide substitution rate estimates (N gene: 1.1×10^−4^ to 3.8×10^−4^ substitutions per site per year, G gene: 5.56×10^−4^ to 1.286×10^−3^ substitutions per site per year) [40], [45], [46], [47], [48]. The age of the MRCA of MOKV was estimated to be 591 years old (95% HPD 294–1005 years) or 657 years old (95%HPD 279–1174 years), respectively. Analyses of the M-gene and P-gene yielded less robust estimates (1883 years and 1703 years respectively) (supplementary material, Fig. S2 and S3) with 95% HPD ranges much wider that the estimates for the N and G gene datasets. This is possibly due to the more variable nature of the M- and P-genes. Estimates based on the concatenated sequences (N, P, M and G-gene coding regions) yielded estimates within the ranges of the other genes (1157 years old, 95%HPD 413–2034 years) (Fig. 3).

**Table 4:** Results from molecular clock analysis for all genes.

Gene analyzed	Age of MOKV MRCA	Nucleotide substitution rate (substitutions/site/year)
N gene	591 yrs (294–1005 yrs)	2.516E^−4^ (1.2009E^−4^ to 3.8862E^−4^)
P gene	1703 yrs (214–3907 yrs)	1.114E^−4^ (2.0828E^−5^ to 2.5012E^−4^)
M gene	1883 yrs (392–4318 yrs)	7.963E^−5^ (1.4543E^−5^ to 1.698E^−4^)
G gene	657 yrs (279–1174 yrs)	2.123E^−4^ (5.5067E^−5^ to 3.7878E^−4^)

Ninety five percent HPD values are indicated in brackets.

The successful use of NGS on four of the isolates (RV4, RV1017, RV1021 and RV1035) enabled full genome consensus sequences to be obtained without the use of specific primers. In comparison to the work involved to obtain gene specific sequences for N, P, M and G on each of the MOKV isolates this approach was relatively simple and time efficient. Of the total number of reads obtained from the brain RNA preparations, between 0.25 and 1% were viral equating to between 294 and 1006 reads.

## Discussion

This study was aimed at producing further insights into the phylogeny and diversity within a unique African lyssavirus species, MOKV. It was our objective to include all MOKV's encountered in history, but the existence or identity of several reported viruses and/or isolates could not be corroborated (Table 1). These included 3 virus isolates that were reported from Nigeria, the existence of which is now doubtful [6], [8], [9] and an isolate reported recently from South Africa [20]. It was also unfortunate that isolates from Cameroon and Ethiopia had to be excluded from this study, as these viruses, in our hands, were likely of the same original stock.

Nevertheless, a panel of 18 MOKV isolated over a period of nearly 50 years (Table 1) could be included and thus represents the most comprehensive phylogenetic analysis of full N, P, M and G genes of the MOKV species.

The monophyletic grouping of isolates from the KZN province of South Africa, isolated over a period of 28 years, indicates the continual presence and stability of the same viral lineage in this geographical domain. This KZN group could be distinguished from the other South African MOKV group, from the EC province, but the time point of divergence is rather recent with the MCRA for these two MOKV groups in the order of 150 years. The sequence diversity observed also seems to determine biological properties of the isolates.

Parallel experimental infection studies in mice showed that the pathogenicity of MOKV (isolates 12341, 252/97, Table 1) had been underestimated, although specific markers could not be determined [49]. Our analysis using a more comprehensive set of sequences corroborated these results (Table 2), but the relevance need to be confirmed by further studies.

Previously, when lyssaviruses were still classified according to genotypes, it was proposed that a new genotype is defined by >80% nucleotide differences and >92–93% amino acid differences [50], [51]. Although this classification is no longer used, the p-distance analysis in this study indicated the MOKV isolates fall within the defined ranges. The MOKV isolates also displayed less sequence divergence than that seen among LBV isolates (20.9% nucleotide sequence difference and 6.7% amino acid sequence difference between LBV isolates). However, the delineation between LBV and MOKV using maximum clade credibility appears not as robust as when using M-gene where LBV isolates rather cluster with MOKV (supplementary material, Fig. S3).

MRCA estimates of MOKV utilizing different genes ranged widely from 591 to 1883 years (HPD 214–4318 years). Although these dates for the MOKV MRCA correspond with the timeframe estimated by Bourhy et al. [47] for the emergence of RABV associated with non-flying mammals (749 years ago, 95% HPD 363–1215 years), it must be noted that the small sample size of MOKV could also influence the robustness of the results. Also, purifying selection can mask the ancient age of viruses that appear to have recent origins as shown for other RNA-viruses [52], [53], thus making it difficult to objectively model the evolutionary history of MOKV.

Use of NGS technologies to obtain four of the MOKV genomes directly from RNA preparations without amplification using specific primers was highly successful. Unlike the approach taken for the other isolates where often primers had to be designed for each specific isolate due to the high divergence seen in the sequences between the MOKV viruses, for the NGS approach the same methodology, i.e. random priming was applied to all isolates, regardless of their divergence. Although a high percentage of non-target sequences were produced, full coding sequences were obtained from all four MOKV isolates which was then put forward to the individual gene analyses. Given the inherent accuracy of 99.9% of Roche 454 and a sequence depth between 294 and 1006 reads per position the inclusion of sequencing errors are highly unlikely.

Twenty three MOKV isolations and one PCR-based identification have been made to date from six African countries in a period of more than 40 years. Since these African countries are from regions in Africa that are far apart, it is likely that MOKV is present in many others African countries and spread over vast portions of the continent. Moreover, over the past almost 20 years MOKV has only been isolated from South Africa (Fig. 1). Since it is known that MOKV is not only limited to South Africa, the lack of isolation from elsewhere is reflective of the non-existence of appropriate surveillance, including for rabies virus, across Africa. Limited diagnostic capabilities (e.g. typing or sequencing of rabies cases/specimens/isolates) across the continent, remains a key factor. Such enhanced surveillance would likely result in the discovery of more isolates and therefore, a higher diversity of MOKV and would thus improve our understanding of MOKV incidence and circulation. Since rabies vaccines do not offer protection against MOKV, a case can be made for the relative importance of a better understanding of the ecology of MOKV [32]. One of the limiting factors in studying MOKV is the fact that the reservoir species for this virus is not known. Although shrews have been implicated, it remains speculative. Lyssaviruses have a strong association with bats and it seems peculiar that MOKV may be the only exception in this regard - among all the other members of the genus. Indeed, virus neutralizing antibodies (VNA), neutralizing both LBV and MOKV have been detected in sera from frugivorous bats (*Rousettus aegyptiacus* and *Eidolon helvum*) [54], [55], [56]. However, belonging to the same phylogroup II, LBV and MOKV have been reported to cross react in serological assays [7], [24], [45], [57]. Since there have been repeated reports of LBV isolations from fruit bats [58], [59], the neutralizing activity of bat sera to MOKV apparently does not confirm the circulation of MOKV in those bat species. However, it cannot be excluded that other yet unidentified African bats may act as reservoir for MOKV. On the other hand, consistent encounters of MOKV in domestic cats and small mammalian species invite speculation along the lines of a prey-to-predator transmission pathway. For MOKV, the estimated MRCA from our study coincides and provides support for the timeframe suggested for the emergence of terrestrial rabies [47]. It is possible that MOKV remained stable in an extant African host environment, while RABV evolution was vastly accelerated given a plethora of host opportunities and global distribution.

## Supporting Information

Figure S1 MCC phylogenetic tree based on the entire nucleotide sequence of the N gene of MOKV isolates and representative lyssavirus isolates.
(EPS)

Figure S2 MCC phylogenetic tree based on the entire nucleotide sequences of the P gene of MOKV isolates and representative lyssavirus isolates.
(EPS)

Figure S3 MCC phylogenetic tree based on the entire nucleotide sequences of the M gene of MOKV isolates and representative lyssavirus isolates.
(EPS)

Figure S4 MCC phylogenetic tree based on the entire nucleotide sequences of the G gene of MOKV isolates and representative lyssavirus isolates.
(EPS)

Table S1 Additional lyssavirus sequences used in phylogenetic analyses (see figure 2–3 and figure S1-S4).
(DOCX)
