# Supplementary material for: Diversity and Epidemiology of Mokola Virus
Source: PLoS Negl Trop Dis. 2013 Oct 24;7(10):e2511. doi: 10.1371/journal.pntd.0002511 (PMC3812115; doi:10.1371/journal.pntd.0002511)
Supplement: Table S1 — Additional lyssavirus sequences used in phylogenetic analyses (see figure 2–3 and figure S1-S4). (DOCX) [file pntd.0002511.s005.docx]

**Diversity and epidemiology of Mokola virus**

J. Kgaladi^1^, N. Wright^1^, J. Coertse^1^, W. Markotter^1^, D. Marston^2^, A. R. Fooks^2,5^, C. M. Freuling^3^, T. F. Müller^3*^, C. T. Sabeta^4^ and L. H. Nel^1^

**Supplementary material**

Table S1: Additional lyssavirus isolates used in phylogenetic analysis (see figure 2-3 and figure S 1-4).

|  | | | |  | |  | |  |
| --- | --- | --- | --- | --- | --- | --- | --- | --- |
| **Lyssavirus species** | **Lab reference number** | **Host species/vector** | **Origin** | | **Year** | | **Accession numbers** | |
| RABV | PV | Vaccine |  | | 1882 | | NC_001542 | |
| RABV | Nishigahara |  | Japan | | 1915 | | AB044824 | |
| RABV | RC-HL |  | Japan | | 1918 | | AB009663 | |
| RABV | HEP-Flury |  | USA | | 1939 | | AB085828 | |
| RABV | 8743THA | Human | Thailand | | 1983 | | EU293121 | |
| RABV | 8764THA | Human | Thailand | | 1983 | | EU293111 | |
| RABV | SHBRV-18 | *Lasionycteris noctivaganas* | USA | | 1983 | | AY705373 | |
| RABV | SAD B19 | Vaccine |  | | 1990 | | M31046 | |
| RABV | 9001FRA | Canine | French Guyana | | 1990 | | EU293113 | |
| RABV | 9147FRA | Fox | France | | 1991 | | EU293115 | |
| RABV | 9704ARG | *Tadarida brasiliensis* | Argentina | | 1997 | | EU293116 | |
| RABV | RABV | Human | India | | 2004 | | AY956319 | |
| RABV | NNV-RAB-H | Human | India | | 2006 | | EF437215 | |
| LBV | LBVNIG1956 | *Eidolon helvum* | Nigeria | | 1956 | | EF547459 (N); EF547407 (P); EF547444 (M); EF547431 (G) | |
| LBV | LBVCAR1974 | *Micropteropus pusillus* | Central African Republic | | 1974 | | EF547449 (N); EF547417 (P); EF547443 (M); EF547430 (G) | |
| LBV | LBVSA1982 | *Epomophorus wahlbergi* | South Africa | | 1982 | | EF547455 (N); EF547410 (P); EF547439 (M); EF547425 (G) | |
| LBV | LBVSEN1985 | *E. helvum* | Senegal | | 1985 | | EF547448 (N); EF547419 (P); EF547446 (M); EF547433 (G) | |
| LBV | LBVZIM1986 | Feline | Zimbabwe | | 1986 | | EF547450 (N); EF547416 (P); EF547442 (M); EF547429 (G) | |
| LBV | LBVSA1981(640) | *E. wahlbergi* | South Africa | | 1980-1981 | | EF547457 (N); EF547408 (P); EF547436 (M); EF547427 (G) | |
| LBV | LBVSA1981(1248) | *E. wahlbergi* | South Africa | | 1980-1981 | | EF547456 (N); EF547412 (P); EF547437 (M); EF547426 (G) | |
| LBV | LBVSA1981(679) | *E. wahlbergi* | South Africa | | 1980-1981 | | EF547454 (N); EF547411 (P); EF547441 (M); EF547424 (G) | |
| LBV | LBVAFR1999 | *Rousettus aegyptiacus* | Egypt/Togo | | 1999 | | EF547447 (N); EF547418 (P); EF547445 (M); EF547432 (G) | |
| LBV | LagSA2003 | *E. wahlbergi* | South Africa | | 2003 | | EF547451 (N); EF547413 (P); EF547434 (M); EF547421 (G) | |
| LBV | LagSA2004 | *E. wahlbergi* | South Africa | | 2004 | | EF547458 (N); EF547415 (P); EF547440 (M); EF547428 (G) | |
| LBV | Mongoose2004 | Mongoose | South Africa | | 2004 | | EF547453 (N); EF547409 (P); EF547438 (M); EF547423 (G) | |
| LBV | LBVSA2006 | *E. wahlbergi* | South Africa | | 2006 | | EF547452 (N); EF547414 (P); EF547435 (M); EF547422 (G) | |
| LBV | LBVSA2008 | *E. wahlbergi* | South Africa | | 2008 | | HM179509 (N); HQ266634 (P); HQ266612 (M); HQ266623 (G) | |
| LBV | KE576 | *R. aegyptiacus* | Kenya | | 2008 | | GU170202 | |
| SHIBV | SHIBV | *Hipposideros commersoni* | Kenya | | 2009 | | GU170201 | |
| DUVV | 86132SA | Human | South Africa | | 1971 | | EU293119 | |
| DUVV | 94286SA | *Minopterus spp* | South Africa | | 1981 | | EU293120 | |
| European bat lyssavirus 1  (EBLV-1) | RV9 | *Eptesicus serotinus* | Germany | | 1968 | | EF157976 | |
| EBLV-1 | 8918FRA | *E. serotinus* | France | | 1989 | | EU293112 | |
| EBLV-1 | 03002FRA | *E. serotinus* | France | | 2003 | | EU293109 | |
| European bat lyssavirus 2  (EBLV-2) | 9018HOL | *Mytosis dasycneme* | Holand | | 1986 | | EU293114 | |
| EBLV-2 | RV1333 | Human | Scotland | | 2002 | | EF157977 | |
| Australian bat lyssavirus  (ABLV) | ABLh | Human | Australia | | 1986 | | AF418014 | |
| ABLV | ABLh | *Pteropus spp.* | Australia | | 1996 | | NC_003243 | |
| ABLV |  |  | Australia | | 1996 | | AF006497 | |
| Khujand virus (KHUV) | KHUV | *Myotis mystacinus* | Tajikistan | | 2001 | | EF614261 | |
| West Caucasian bat virus  (WCBV) | WCBV | *Miniopterus schreibersii* | Russia | | 2002 | | EF614258 | |
| Aravan virus (ARAV) | ARAV | *Myotis blythi* | Kyrghyzstan | | 1991 | | EF614259 | |
| Irkut virus (IRKV) | IRKV | *Murina leucogaster* | Russia | | 2002 | | EF614260/ AY333112 | |
| IRKV | IRKV | *Murina leucogaster* | China | | 2012 | | JX442979 | |
| IRKV | IRKV | *Human* | Russia | | 2007 | | FJ905105 | |
| Bokeloh bat lyssavirus  (BBLV) | 21969 | *Myotis nattererii* | Germany | | 2010 | | JF311903 | |
| Ikoma virus (IKOV) | RV2508 | *Civettictis civetta* | Tansania | | 2009 | | JX193798 | |
